# Phage proteins are expressed on the surface of *Neisseria gonorrhoeae* and are potential vaccine candidates

**DOI:** 10.1371/journal.pone.0202437

**Published:** 2018-08-23

**Authors:** Aneta Kłyż, Andrzej Piekarowicz

**Affiliations:** Department of Virology, Institute of Microbiology, Faculty of Biology, University of Warsaw, Warsaw, Poland; Emory University School of Medicine, UNITED STATES

## Abstract

All *Neisseria gonorrhoeae* strains whose DNA sequences have been determined possess filamentous phage sequences representing their full genomes. The presence of filamentous phage DNA sequences in all sequenced *N*. *gonorrhoeae* strains suggest that purified phage particles might be used as a gonococcal vaccine. To test this hypothesis, we purified filamentous NgoΦfil phages and immunized rabbits subcutaneously. The elicited sera contained large quantities of anti-phage IgG and IgA antibodies that bound to the surface of *N*. *gonorrhoeae* cells, as shown by ELISA and flow cytometry. The elicited sera bound to the structural NgoΦ6fil proteins present in phage particles and to *N*. *gonorrhoeae* cells. The sera did not react with gonococcal outer membrane proteins. The sera also had bactericidal activity and blocked adhesion of gonococci to tissue culture cells. These data demonstrate that NgoΦfil phage particles can induce antibodies with anti-gonococcal activity and may be a candidate for vaccine development.

## Introduction

Vaccination is one of the basic methods of prevention against bacterial and viral diseases in humans and animals and plays an important role in the eradication of several of them [1, 2]. A good vaccine must to contain antigens in the form present in native infectious agent [1,2]. There are several common types of vaccines that align with these criteria, such as (1) live attenuated microorganisms, (2) protein subunits of microorganisms, (3) recombinant bacteria or viruses expressing foreign antigens and finally, (4) DNA vaccines coding for specific antigens produced after entry of DNA into the host organism and expression of encoded proteins [1, 2, 3, 4].

Filamentous bacteriophages broadly used in different aspects of phage display technology are also used in the construction of vaccines as vectors carrying foreign antigens [5–19]. However, they have never been used directly as an antigen in the construction of a vaccine with one exception, when *Salmonella enterica* ser. Typhimurium carrying phagemid pBS::Φ6fm) was used to test for production antibodies after oral administration into rabbits [20]. The sera produced by vaccinated rabbit’s elicited large amounts of IgG and IgA antibodies, were bound by *N*. *gonorrhoeae* cells and were able to kill the cells [20]. This represents a novel approach to the problem of constructing a vaccine against *N*. *gonorrhoeae* [20]. All attempts to construct such a vaccine based on different gonococcal proteins and molecules have not produced fully satisfying results [21].

Attempts to construct a vaccine against *N*. *gonorrhoeae* using filamentous phages produced by these bacteria are based on the presence of DNA sequences encoding these phages in all *N*. *gonorrhoeae* strains tested so far [22–24] and the degree of conservation of their DNA and protein sequences approaching 95% [22–24]. There are four genetic islands in *N gonorrhoeae* encoding four filamentous phages, NgoΦ6, NgoΦ7, NgoΦ8 and NgoΦ9 [22–24]. The first two are able to produce fully infective phages [23, 24], and produced phages can be formed from proteins of all four phages. The *N*. *gonorrhoeae* phages NgoΦ6 and NgoΦ7 share homology with the group of phages able to integrate into the host chromosome [22, 23]. The genetic organization of the NgoΦ6 and NgoΦ7 phage genomes possess the same blocks of genes responsible for phage functions [22–24]. Among these blocks, it was predicted that the genes *orf*3 to *orf*6 are responsible for coding small structural proteins, *orf*7 is the gene coding for a protein of 58 kDa that is a homolog of a filamentous phages protein responsible for adsorption to the host cell and the start of phage infection, and *orf9* is the gene responsible for phage extrusion [22–24]. All these proteins should be present on the surface of gonococcal cells during the assembly and release of phage particles, making them good vaccine candidates. In this paper, our results show that purified wild-type filamentous NgoΦfil phage particles delivered via the subcutaneous route elicit bactericidal antibodies.

## Results

### Proteins present in preparations of NgoΦfil filamentous phages

*N*. *gonorrhoeae* possesses four genetic islands encoding genes sharing similarity with representatives of *Inoviridae* phages [21–24]. Two of them, designated NgoΦ6 and NgoΦ7, encode genes necessary for biological activity such as the ability to produce progeny phages [22–24], while NgoΦ8 lacks genes, and NgoΦ9 represents a truncated form of the phage genome [22–24]. The presumptive main structural proteins of NgoΦ6 and NgoΦ7 (ORF4 and ORF5) share over 95% identity in their amino acid sequences [22, 23]. The filamentous phages produced by *N*. *gonorrhoeae* are a mixture of NgoΦ6, NgoΦ7 and NgoΦ8 [22, 23] (named together as NgoΦfil), and their proteins can be a mixture of the gene products of all four islands. Among the 11 annotated open reading frames found in NgoΦ6 in the FA1090 genome (GenBank accession number AE004969.1), the genes *orf3*, *orf4*, *orf5*, *orf6* and *orf7* are responsible for the formation of structural proteins with molecular sizes of 7.7 kDa, 12.5 kDa, 12.0 kDa, 12.5 kDa and 58 kDa [22, 23], respectively. The main structural proteins of filamentous phage MDAΦ of *Neisseria meningitidis*, which is closely related to phage NgoΦ6 [25], are formed by two proteins, ORF4 and ORF5, corresponding to ORF4 and ORF5 of NgoΦfil phages of *N*. *gonorrhoeae*. To test this prediction, we constructed the pBS::NgoΦ6fm phagemid containing the ORF5 protein fused with the FLAG epitope at its 5’ C-terminus. Two proteins derived from this phagemid particle were detected by western blotting with monoclonal anti-FLAG IgG antibody reactivity with two proteins of 12.5 kDa and 25 KDa (S1 Fig). The larger is formed by a fusion of protein ORF4 and ORF5 as predicted by analysis of the DNA sequence of the region encoding ORF4, and the smaller is the ORF5 protein.

**Fig 1:**
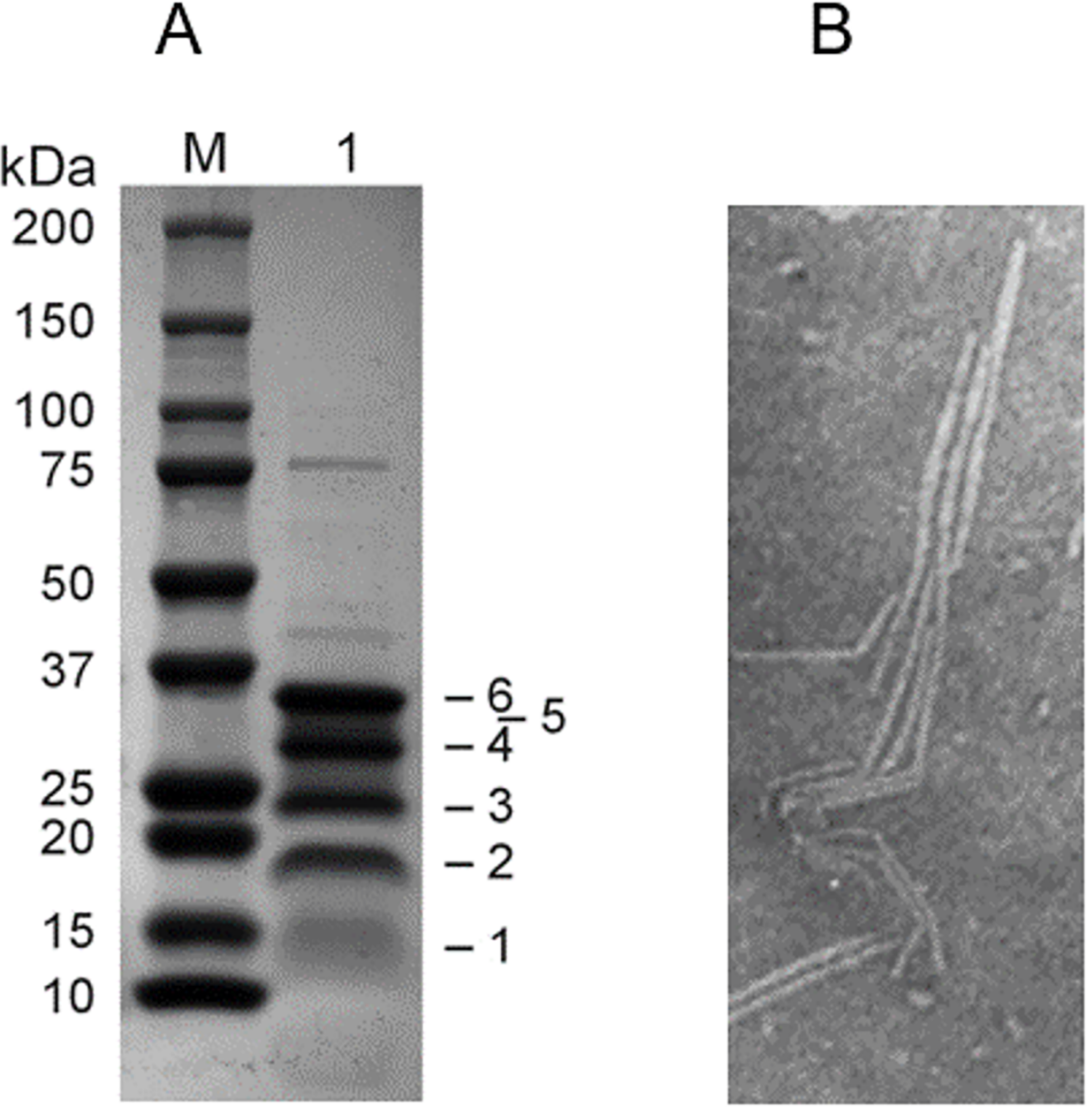
SDS-PAGE profile of proteins present in NgoΦfil phage particles propagated in *N*. *gonorrhoeae* cells. Phage particles were purified as described in the Materials and Methods, and proteins were separated on a 5–15% gradient gel. Panel A; Lane M; molecular weight standard. Lane 1; phage proteins. Numbers on the right (1 and 3; 12.5 kDa and 25 kDa) indicate phage-encoded structural proteins found in the phage particle preparation, while numbers 2, 4, 5 and 6 indicate host outer membrane proteins of molecular sizes 18 kDa, 30 kDa, 32 kDa and 35 kDa respectively. Several additional proteins are also visible. (For the original gel, see S1 Fig). Panel B; Transmission electron micrograph of NgoΦ6fil isolated from *N*. *gonorrhoeae* FA1090 cells. Culture supernatants were precipitated with PEG 8000 and NaCl, purified on Sephacell QMA columns, dialyzed against TE buffer, added to a gold grid, stained with uranyl acetate, and visualized on a Zeiss EM10CA electron microscope.

NgoΦfil phages used for rabbit vaccination were purified by the previously described method that showed by EM the presence of phage particles without visible nonspecific material (Fig 1). SDS-PAGE analysis of proteins from purified filamentous phages isolated from the culture of *N*. *gonorrhoeae* FA1090 (Fig 1) showed the presence of phage-encoded proteins of 12.0 kDa and 12.5 kDa, which correspond to the predicted structural phage proteins and a fused protein of 24.5 kDa as well as several host proteins ranging in molecular size from 18 kDa to 75 kDa.

### NgoΦfil phages elicit antibodies reactive with NgoΦfil phage particles

**Fig 2:**
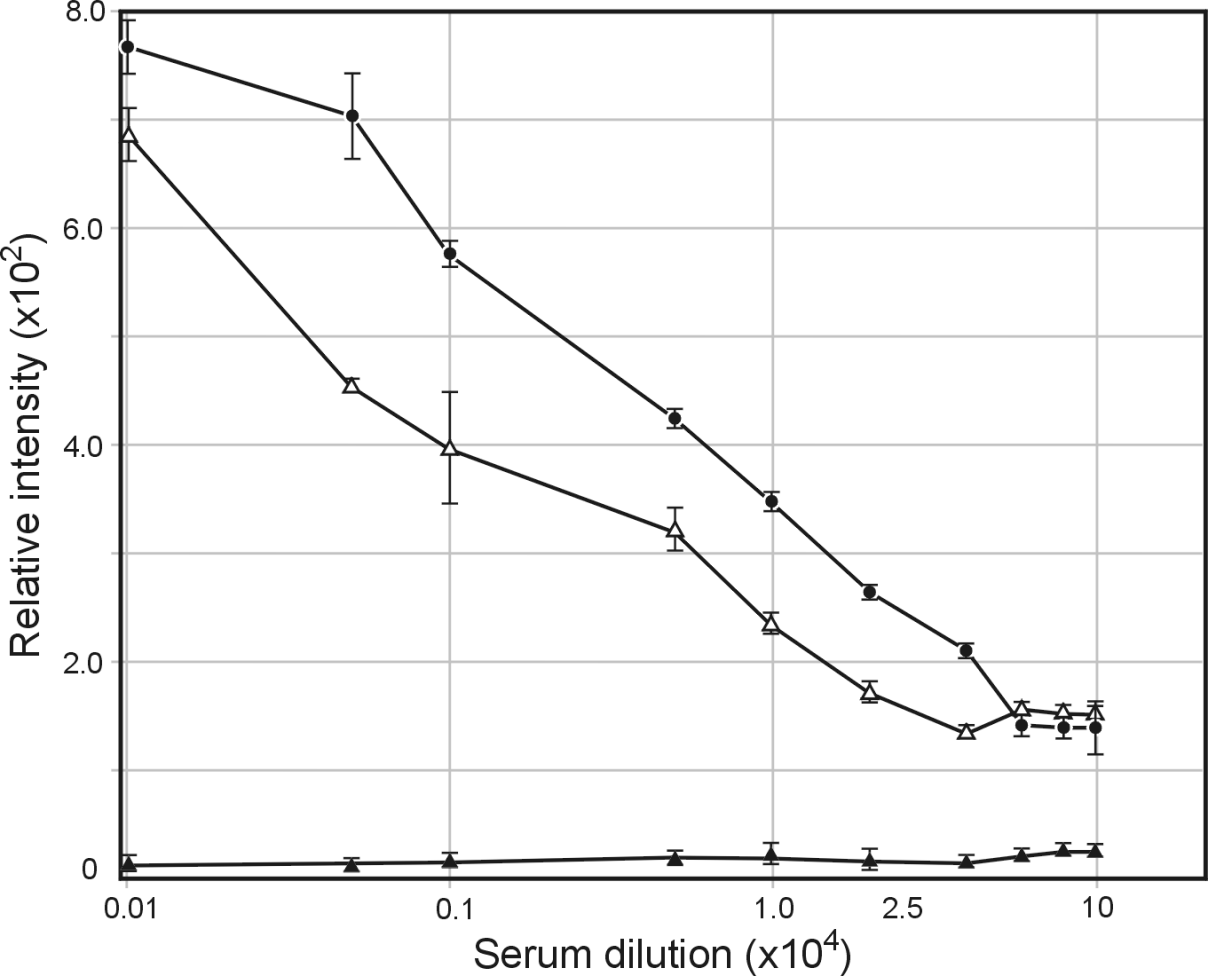
Serum IgG antibody levels specific for *N*. *gonorrhoeae* phage NgoΦfil. Rabbits were immunized subcutaneously with purified NgoΦfil. The sera obtained at days 14 and 28 were analyzed by quantitative dot ELISA. NgoΦfil phage particles (an equivalent of 3 μg of protein) were affixed to nitrocellulose filters and incubated with different dilutions of rabbit sera. Binding of anti-phage NgoΦfil IgG was detected with goat anti-rabbit IgG-alkaline phosphatase conjugate. Data are presented as the mean ± S.D. of two separate experiments each performed in duplicate. The intensity of the color of each spot was expressed as the change in spot intensity compared to the negative control where spotting of NgoΦfil was omitted. For each point, 4 spots were analyzed. The lanes represent the following: ▲-▲, pre-immunized sera, Δ—Δ, day 14 sera, ● ●-; day 28 sera. One-tailed *P* values of ≤ 0.10 for day 14 and 28 sera were considered statistically significant.

Previously, we have shown that rabbits orally infected with *S*. *enterica* ser. Typhimurium strain #3987 harboring phagemid pBS::NgoΦ6fm produces sera containing large quantities of anti-phage IgG and IgA antibodies able to bind to the surface of *N*. *gonorrhoeae* cells [20]. The sera also had bactericidal activity. These antibodies were directed against ORF9, protein that plays a role in the assembly and release of phage particles [22–24]. We wanted to know whether phage structural proteins can serve as antigens in vaccines against *N*. *gonorrhoeae*. These proteins, like ORF9, are present in all so far sequenced *N*. *gonorrhoeae* strains. To determine whether filamentous phages propagated and released from *N*. *gonorrhoeae* cells may serve as an antigen inducing rabbit formation of antibodies recognizing phage particles and *N*. *gonorrhoeae* cells, approximately 200 μg of protein from a phage preparation were used to subcutaneously immunize rabbits without any adjuvant. The quantitative spot ELISA results used for the determination of levels of IgG specific for purified NgoΦfil showed a very high increase after the first immunization and a very low booster effect with additional immunization (Fig 2). As a negative control, we omitted membrane phage particles. As another control, we used pre-immunized rabbit sera that showed a negligible level of activity, and these values were subtracted from the values obtained with immunized sera.

### NgoΦfil phages elicit antibodies reactive with host cells

**Fig 3:**
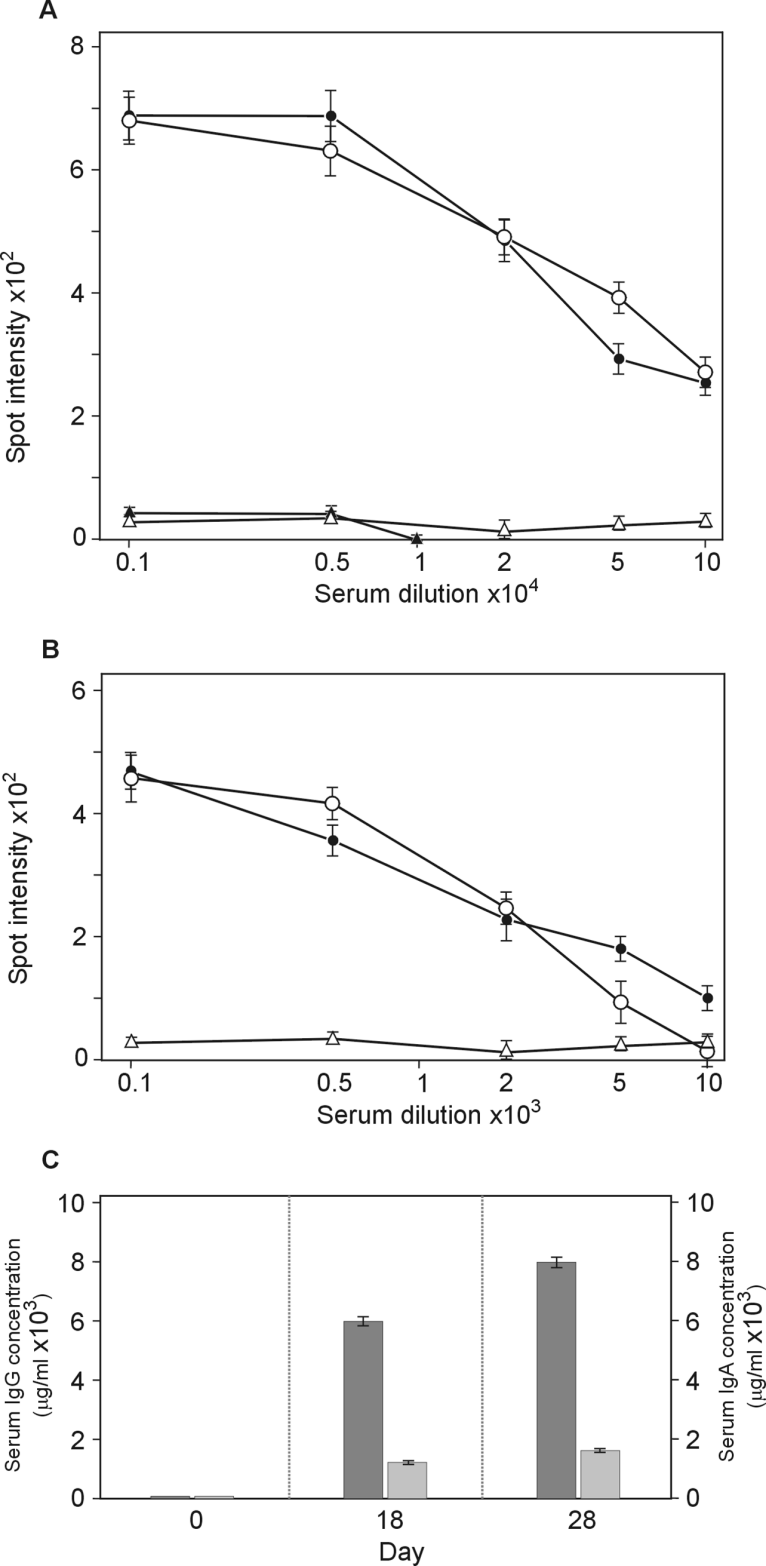
Serum IgG and IgA antibody levels. Sera were analyzed by quantitative dot ELISA. *N*. *gonorrhoeae* cells (3 μl of 10^8^ cells/ml) diluted in PBS were spotted onto a nitrocellulose strip and allowed to dry. Panel (A) Titers of IgG polyclonal antibodies collected on days 0, 14 and 28 after immunization. The intensity of the color of each spot was expressed as the change in spot intensity compared to the negative control where spotting of bacteria was omitted. Binding of anti-phage NgoΦfil IgG was detected with goat anti-rabbit IgG-alkaline phosphatase conjugate. For each point, 4 spots were analyzed. Panel A, Lane: ●—●, day 28; o- o, day 14; Δ–Δ, day 0; lane ▲-▲, day 28 tested with *N*. *gonorrhoeae* FA1090 filamentous phage-deficient cells. Panel (B), titers of IgA polyclonal antibodies. The intensity of the color of each spot was expressed as the change in spot intensity compared to the negative control where spotting of bacteria was omitted. For each point, 4 spots were analyzed. Lane: ● -●, day 28; o- o, day 14; Δ—Δ, day 0). Panel (C) The amount of antibody was determined by comparing the optical density of specific anti-*N*. *gonorrhoeae* antibodies bound to the spots as presented in Panel A and B to a standard curve obtained with known quantities of purified mouse IgG reference antibodies. Standard curves were prepared by quantitative spot ELISA. The amount of protein was quantified using the GeneTools GBox (Syngen) program and expressed as the intensity of the spot versus the concentration of IgG or IgA protein. Since the measurement range of the ELISA dot assay was between dilution 200 and 800, the final determination of IgG concentration in all sera tested was based on spot intensity values in this range. Dark gray rectangle, anti-*N*. *gonorrhoeae* IgG; light gray rectangle, anti-*N*. *gonorrhoeae* IgA. One-tailed *P* values of ≤ 0.05 for day 14 and 28 sera were considered statistically significant.

Because the sera obtained after the immunization of rabbits with purified NgoΦfil particles recognized these particles, we assumed that they would also recognize and bind with *N*. *gonorrhoeae* cells. The same negative controls were used with a quantitative spot ELISA for the determination of IgG and IgA levels in the tested sera. Again, a very high level of IgG antibodies and a lower level of IgA were detected after the first immunization with some booster effect after additional immunization (Fig 3).

The same negative controls were used for the determination of IgG levels versus purified phage particles, where instead of omitting phage particles, bacterial cells were omitted from the membrane. *The N*. *gonorrhoeae* strain lacking all filamentous phage DNA sequences showed the level of reactivity with sera from rabbits immunized with purified NgoΦfil as with pre-immunized sera (Fig 3).

The phage particles used for the immunization of rabbits contained both phage-encoded structural proteins and bacterial proteins. Western blotting was carried out to detect which of these proteins are recognized by antibodies produced after immunization of rabbits with purified phage particles. We expected that both types of proteins, phage and bacterial, should be present in quantities to give a positive signal.

**Fig 4:**
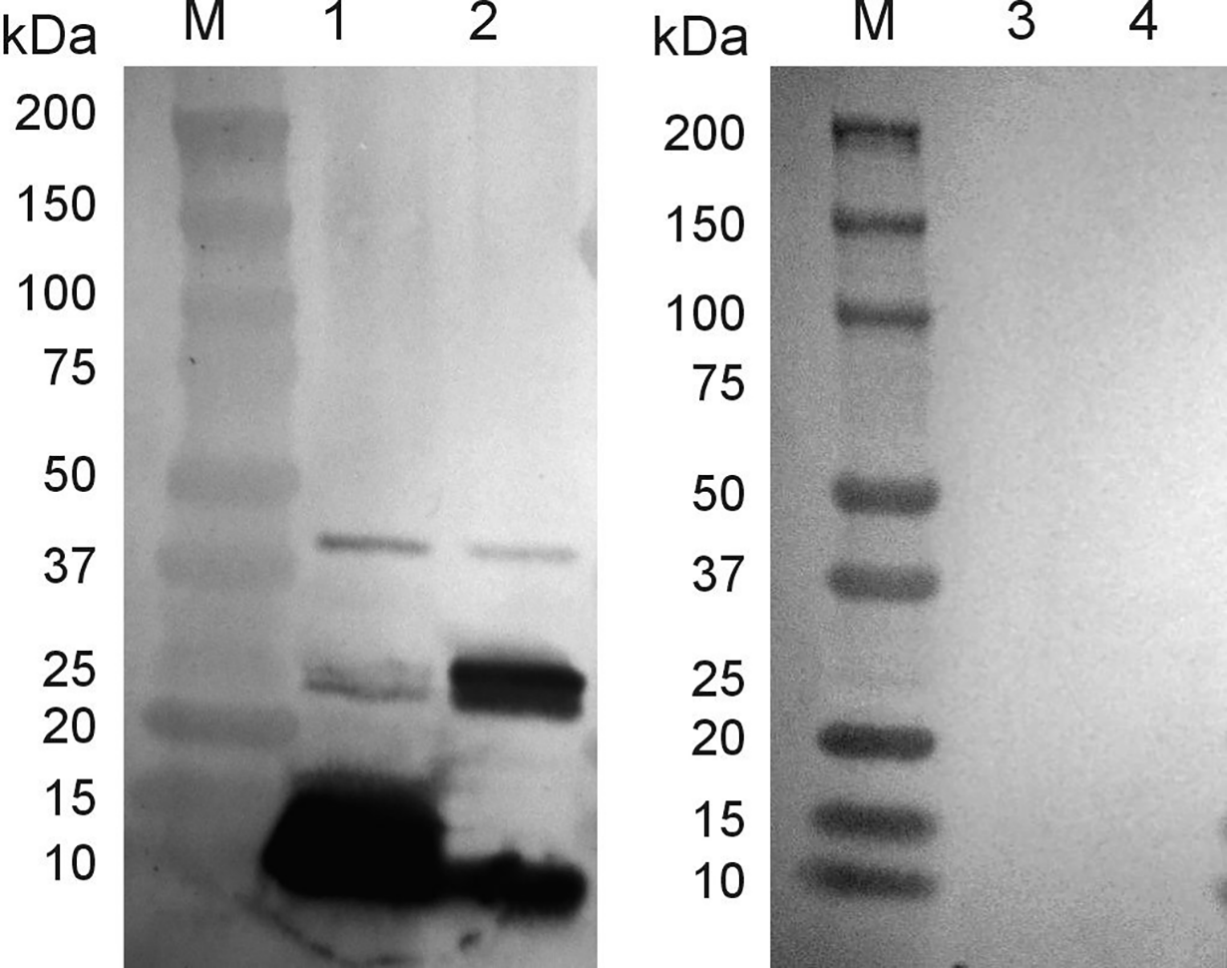
Reactivity of rabbit sera obtained from immunization with *N*. *gonorrhoeae* NgoΦfil phage proteins. Phage particles were separated on SDS-PAGE gels and subjected to Western blot analysis. Lanes 2 and 4, extracts were derived from broth-grown gonococci. In lanes 1 and 3, extracts were from purified phage. Lanes 1 and 2 were incubated with day 28 sera. Lanes 3 and 4 were incubated with pre-immunized rabbit sera. (For original Western blot, see S4 Fig and S5 Fig).

The data in Fig 4 (lane 1) indicate that the elicited antibody formed after the immunization of rabbits with NgoΦfil phages binds only to two proteins present in phage particles with a molecular mass consistent in size with structural phage proteins. Binding to the same two proteins was also observed in *N*. *gonorrhoeae* cell extract (Fig 4, lane 2), although the cell extract showed much more ORF4-ORF5 fused protein. Sera from pre-immunized rabbits did not react with phage proteins (Fig 4, lanes 3 and 4).

### Immunofluorescent staining of *N*. *gonorrhoeae* FA1090

**Fig 5:**
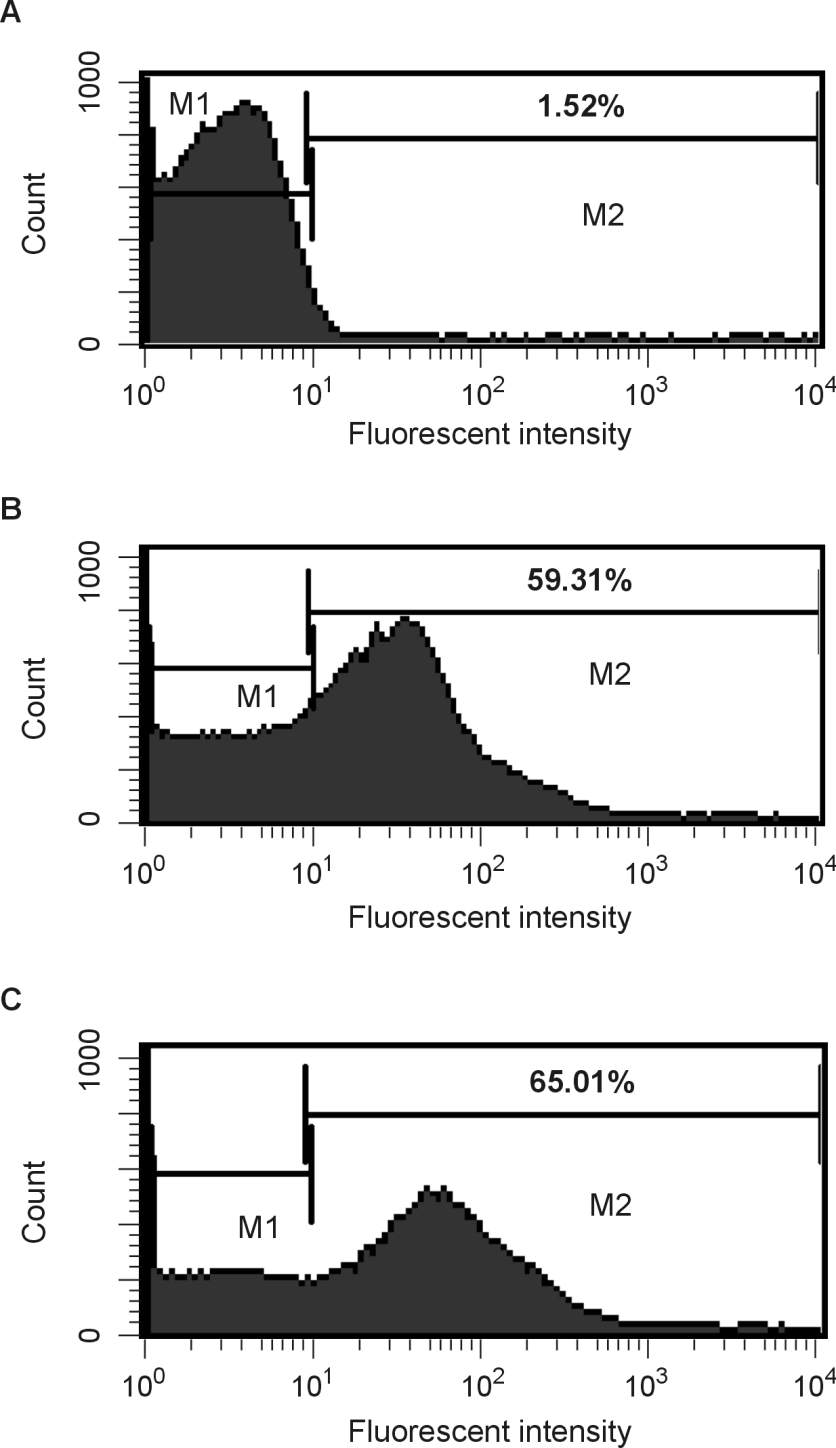
Flow cytometry analysis of antibody binding to *N*. *gonorrhoeae*. FA1090 cells were treated with buffer pre-immunization sera (dilution 1:500) (panel A) or immunized sera (dilution 1:500) obtained after 14 days (panel B) and 28 days (panel C) followed by treatment with Cy3 goat anti-rabbit IgG (Life Technologies). The bacteria were analyzed using a FACSCalibur flow cytometer. Data were analyzed with CellQuest software. Shown are representative histograms from three independent experiments. The bar in the figure represents the gate used to measure binding efficiency. The number in each figure corresponds to the percentage of the population that bound antibody.

The quantification of the percentage of cell-binding antibodies was carried out by flow cytometry. The data in Fig 5 show a significant shift in the binding profile. Using the gating shown in the figure, 65% of the cell population bound significant levels of IgG present in immunized sera (Fig 5B and 5C). The same minimal level of binding was shown with pre-immunized sera (Fig 5A).

### Serum bactericidal activity

**Fig 6:**
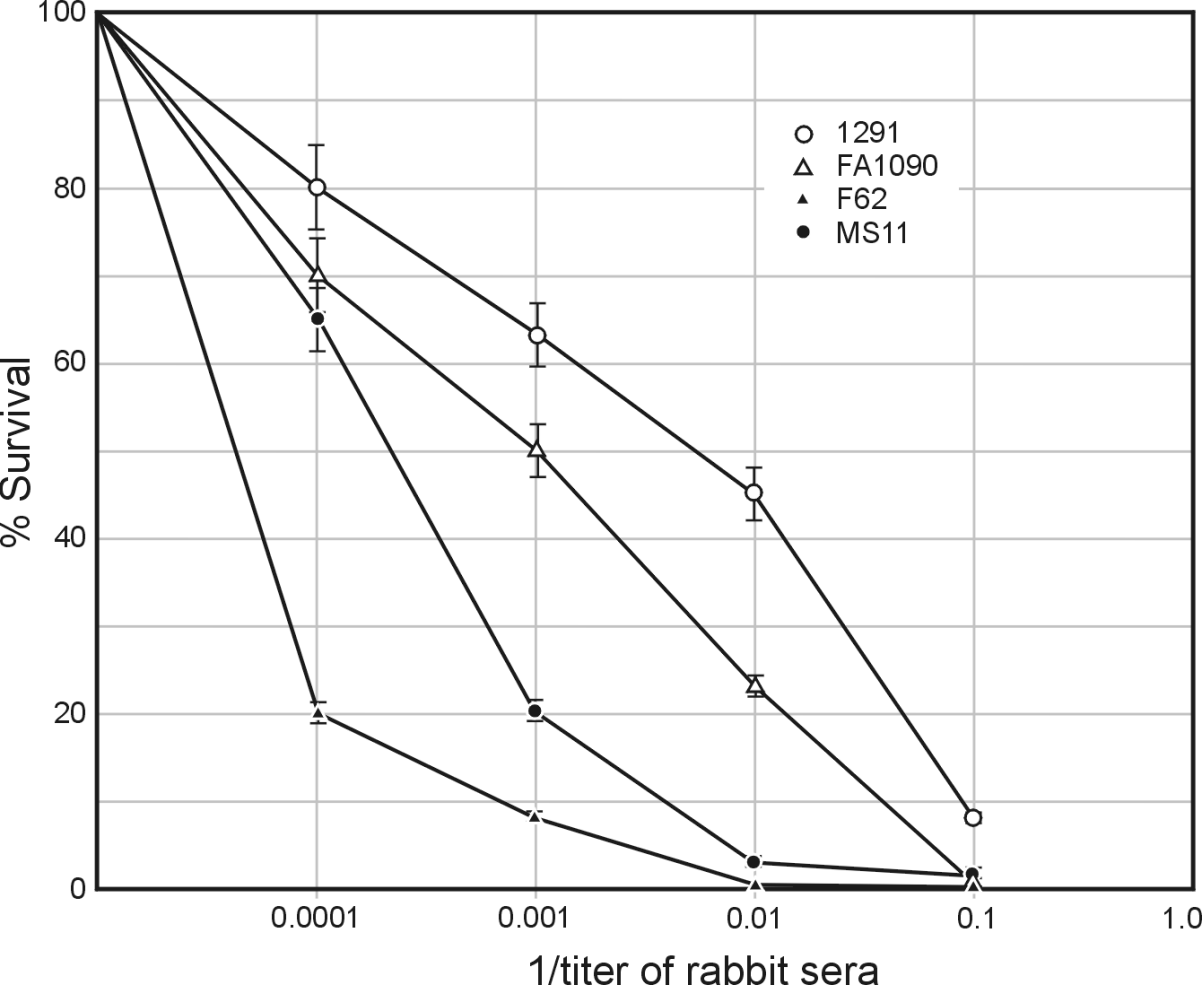
Bactericidal properties of elicited antibody. *N*. *gonorrhoeae*-specific bactericidal activity of sera from immunized rabbits was detected using an antibody complement-mediated bactericidal assay. Serum samples (heat inactivated at 56°C for 30 min.) were mixed with gonococcal cells (3×10^4^ CFU/ml) and incubated at 37°C for 15 min. Normal baby rabbit sera (final concentration of 4%) was added to the mixtures as a complement source and incubated for an additional 45 mins. The number of colony forming units from these mixtures containing the immunized serum were counted and compared to those from negative controls (gonococci incubated with normal rabbit serum). ○—○, *N*. *gonorrhoeae* 1291B; Δ–Δ, *N*. *gonorrhoeae* FA1090; ▲—▲, *N*. *gonorrhoeae* FA62; ●—●, *N*. *gonorrhoeae* MS11; ■—■, *N*. *gonorrhoeae* FA1090 filamentous phage-deficient cells. One-tailed *P* values of ≤ 0.10 for F62 and 1291 B strains and ≤ 0.05 for FA1090 and MS11 strains were considered statistically significant.

The results presented thus far indicate that serum IgG binds native *N*. *gonorrhoeae* bacterial cells. If so, we assumed that sera obtained after immunization of rabbits with NgoΦfil should also elicit bactericidal effects. Using baby rabbit sera as a complement source, we carried out a bacterial killing assay. We used day 28 sera from the rabbits and determined relative bactericidal activities against homologous and heterologous gonococcal strains. We determined that the addition of 8% complement did not result in detectable killing of the three tested strains. The data in Fig 6 indicate that the elicited antibodies were able to kill the three strains to differing degrees while *N*. *gonorrhoeae* strain lacking all filamentous phage DNA sequences was not killed.

### Antibodies block the adhesion of gonococci to eukaryotic cells

**Fig 7:**
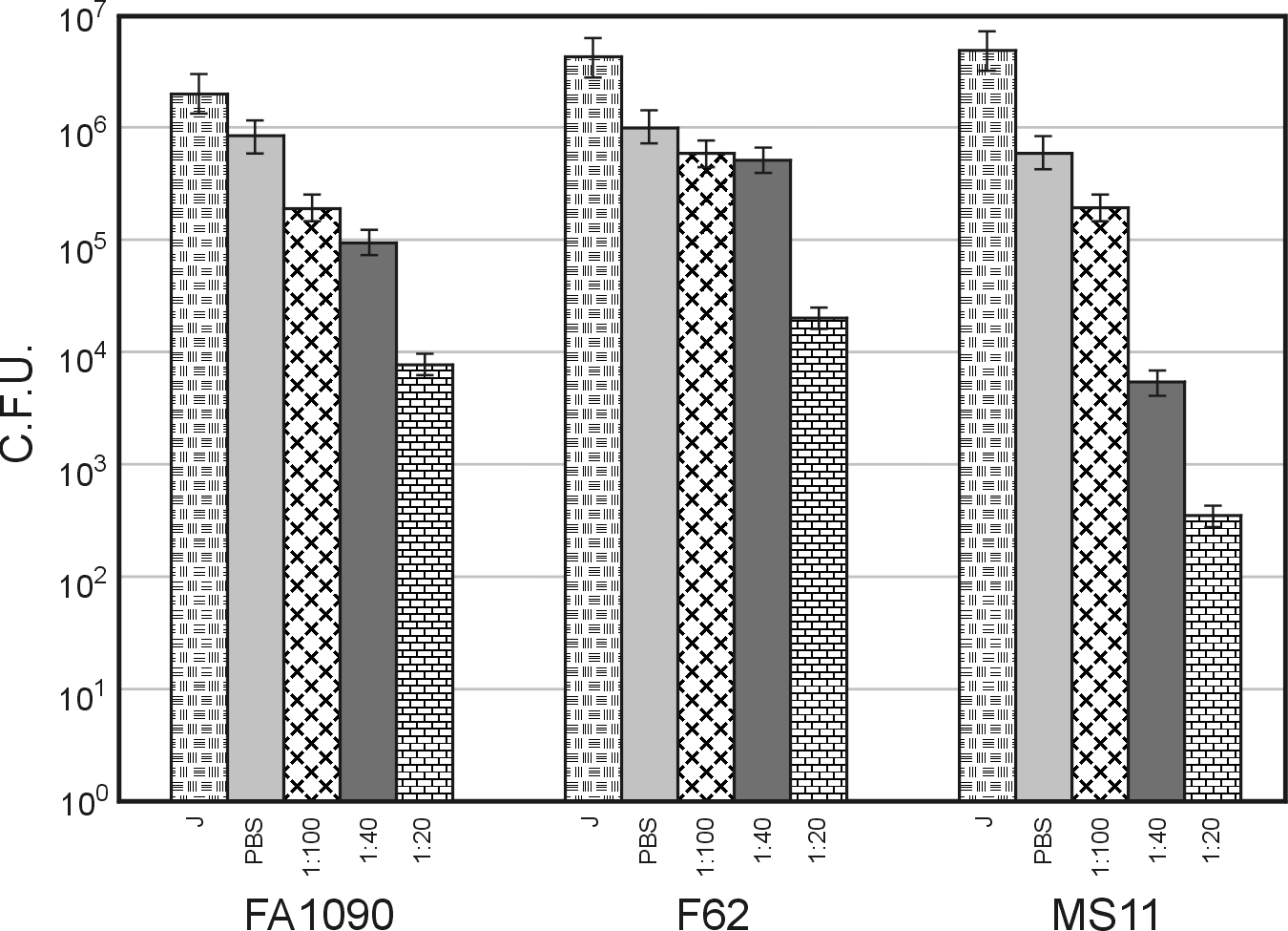
Antibody blocking activity of *in vitro* infection assay with sera produced by immunization of rabbits with NgoΦfil particles. Three dilutions of sera were tested for blocking the binding of different N. gonorrhoeae cells and ME180 cervical epithelial cells (ATCC, Manassas, VA). The data shown represent the geometric means and standard errors of three independent experiments. One-tailed *P* values of ≤ 0.10 for F62 and MS11 strains and ≤ 0.05 for FA1090 and 1291B strains were considered statistically significant.

Antibody-mediated inhibition of adherence may also be protective against gonococcal invasion. To test whether NgoΦfil protein-specific antibodies can block gonococcal interactions with human CEACAM-expressing endocervical cells, ME180 cells were inoculated with antibody-treated bacterial cells. Treatment with day 28 antibodies from bacteria resulted in a dose-dependent decrease in the number of cell-associated bacteria compared to untreated bacteria. In contrast, there was no decrease in the number of cell-associated bacteria when bacteria were treated with unimmunized sera (Fig 7).

### Two types of phage-based anti-*N*. *gonorrhoeae* antibodies elicit different immune responses

**Fig 8:**
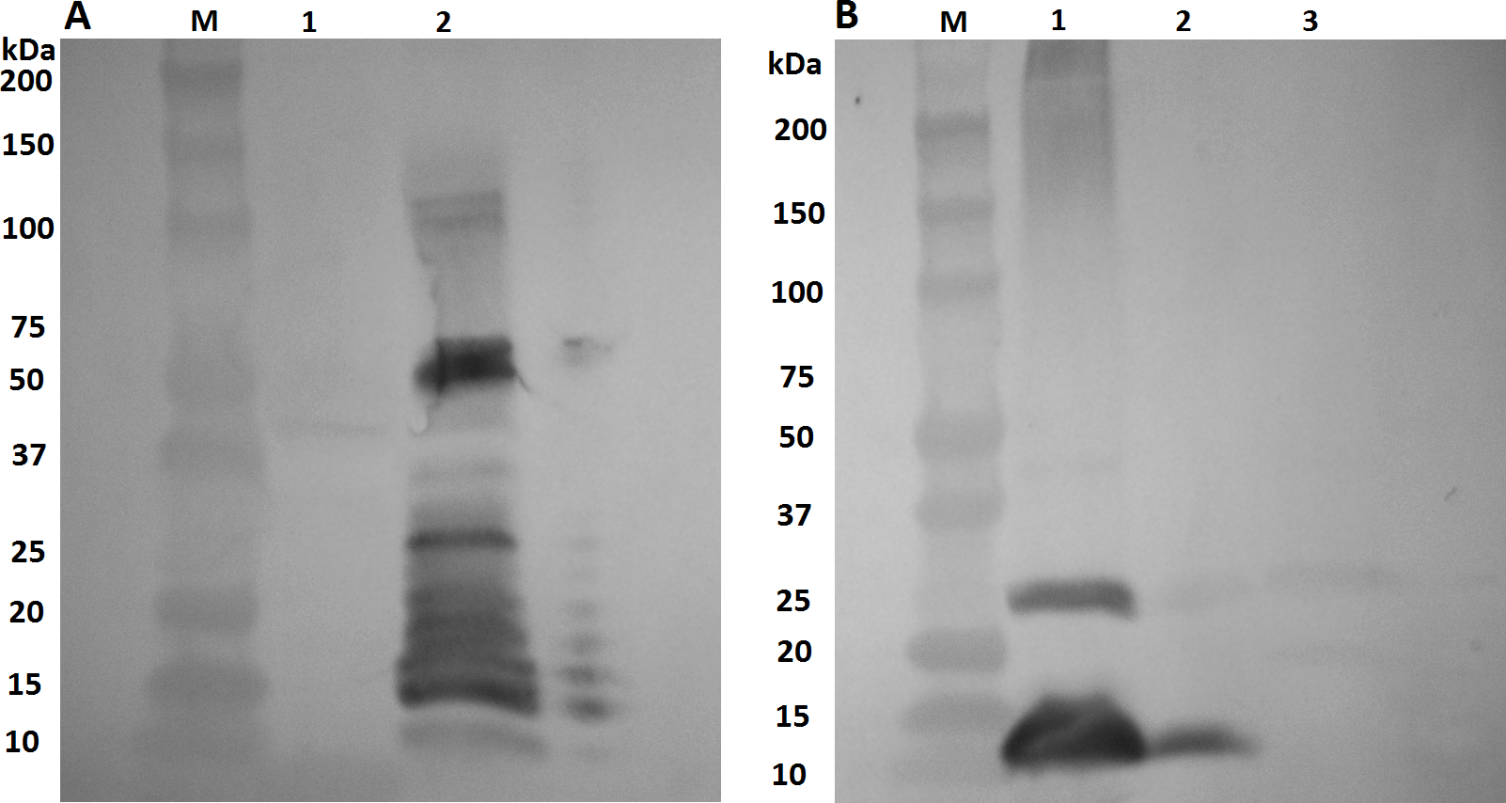
Reactivity of anti-phage NgoΦfil rabbit antibodies with phage structural proteins. Reactivity of two types of rabbit sera obtained after immunization with *S*. *enterica* ser. Typhimurium χ3987 (pBS::NgoΦ6fm) (Panel A) or NgoΦfil phages (Panel B) with NgoΦfil phage proteins (lanes A1 and B1), pBS::NgoΦ6fm phagemid proteins (lanes A2 and B2) and *E*. *coli* Top10 (pBS::NgoΦ6fm) (lane B3). Phage and phagemid particles were separated on SDS-PAGE gels and subjected to Western blot analysis. Lanes A1 and A2 were incubated with day 66 sera, and lanes B1, B2 and B3 were incubated with 28-day rabbit sera.

Two types of sera obtained after immunization of rabbits with *S*. *enterica* ser. Typhimurium (pBS::Φ6fm) (Serum B) and after immunization with NgoΦfil phage particles (Serum A) propagated in *N*. *gonorrhoeae* cells react differently with phage structural proteins. Serum B does not recognize phage structural proteins (ORF4, ORF5, their fusion forms and ORF7) when phages are propagated in *N*. *gonorrhoeae* but recognizes them when phagemid particles are derived from *S*. *enterica* ser. Typhimurium. Serum A reacts in the opposite way, recognizing phage structural proteins present in NgoΦfil phage particles but not in phagemid particles propagated in *Salmonella* cells (Fig 8), suggesting the existence of some type of modification of these proteins.

## Discussion

Considering the dramatically increasing number of gonococcal infections worldwide, it has become necessary to develop an active vaccine against these diseases. To date, attempts to develop vaccines based on different types of cell surface proteins and other molecules have ended without real success [21]. The reasons for this result may be the antigenic variability of surface proteins or other compounds used for the construction of vaccines [21].

The use of filamentous NgoΦ phages as antigens has several advantages; they are present in all so far sequenced strains of *N*. *gonorrhoeae*, and at least three of these phages encode phage structural proteins [22–24]. The presence of these structural proteins decreases the chance of formation of a gonococcal strain deficient in all three phage genomes and indicates a lack of genetic variability in the phages [22–24], and the fact that filamentous phages belong to the *Inovirus* family means that they have very strong adjuvant activity independent of the application route [26]. The level of antibodies elicited after 2–3 immunizations in mice is 1:10^5^ to 1:10^6^ [26].

Previous studies have shown that rabbits immunized with *S*. *enterica* ser. Typhimurium expressing the NgoΦ6 phagemid generate very high levels of antibodies recognizing *N*. *gonorrhoeae* cells [20]. These antibodies recognize only the phage-encoded ORF9 protein that has significant homology with zonula occludes toxin encoded by the CTXΦ phage of *Vibrio cholerae* [27]. We have now shown that purified NgoΦ6 particles administered subcutaneously can elicit formation of very high levels of IgG and slightly lower levels of IgA antibodies. The elicited IgG antibodies showed a strong bactericidal effect and blocking of gonococcal cell adsorption to human cells. The killing of bacteria and blocking of adherence to eukaryotic cells by antibodies depends on the first step of recognition of bacterial cells manifested by their binding to those cells. The Elissa test (Fig 3) showed that *N*. *gonorrhoeae* lacking DNA sequences encoding genomes of filamentous phages are unable to bind antibodies elicited by the immunization of rabbits with purified NgoΦfil particles and are not killed by them (Fig 6). If so we were not expecting that these antibodies would block their adherence to eukaryotic cells.

One of the fundamental differences between the antibodies obtained after immunization of rabbits with *S*. *enterica* ser. Typhimurium (pBS::Φ6fm) (Serum B) and after immunization with NgoΦfil phage particles (Serum A) propagated in *N*. *gonorrhoeae* cells is their different reactivity with phage structural proteins. Serum B does not recognize phage structural proteins ORF4, ORF5, their fusion form or ORF7 when phage is propagated in *N*. *gonorrhoeae* but recognize them when phagemid particles are derived from bacteria such as *S*. *enterica* ser. Typhimurium. Serum A reacts in the opposite way, recognizing phage structural proteins present in NgoΦ6 phage particles but not in phagemid particles propagated in *Salmonella*. These results suggest that phage structural proteins acquire some posttranslational modification during propagation in *N*. *gonorrhoeae* or other bacterial cells that are passively removed during propagation in other bacteria. The presence of post-translational protein modification in bacteria and its influence on bacterial physiology is now well established [28].

Subcutaneous immunization with NgoΦfil does not remove this modification from phage structural proteins present in phage particles, and as result, antibodies elicited by subcutaneous immunization with NgoΦfil phage particles specifically recognize only phage structural proteins possessing such modification. On the other hand, it seems that similar post-translational modification of host proteins present in phage particles is lost after subcutaneous immunization because elicited antibodies against them do not react with *N*. *gonorrhoeae* proteins present in phage particles. This type of antigenic specificity acquired through posttranslational modification of proteins in *N*. *gonorrhoeae* cells may influence the activity of anti-*N*. *gonorrhoeae* vaccines based on gonococci proteins produced in bacteria other than native strains.

Propagation of NgoΦ6 phage particles in *N*. *gonorrhoeae* cells with the aim of using them to create a vaccine can be difficult and would require special precautions due to the strong pathogenicity of these bacteria. Preliminary results showing strong reactivity of pBS::NgoΦ6fm phagemid particles propagated in *H*. *influenzae* with antibodies elicited by immunization of rabbits with NgoΦfil phage particles suggest that phagemid particles propagated in *H*. *influenzae* could elicit antibodies against *N*. *gonorrhoeae* with the same reactivity as phage propagated in *N*. *gonorrhoeae*. Production of phagemid particles in *H*. *influenzae* would be cheaper and would not require safety measures as strong as those for phage propagated in *N*. *gonorrhoeae*. An alternative would be to use a *Salmonella* strain carrying pBS::Φ6fm that would induce antibodies recognizing both the ORF9 protein and structural proteins of the NgoΦ6 phage.

Our paper describes the direct use of filamentous phage as a potential vaccine against gonococci. Altogether, our results demonstrated that Ngo::Φ6fm can serve as an efficient antigen system and has the potential to form the basis for a vaccine against *N*. *gonorrhoeae*.

## Materials and methods

### Bacterial strains, plasmids, phages, and growth conditions

*Escherichia coli* strain DH5α, F− φ80 *lac*ZΔM15 Δ(*lac*ZYA-*arg*F)U169 *rec*A1 *end*A1 *hsd*R17(*r*K− *m*K+) *pho*A *sup*E44 λ-*thi*-1 *gyr*A96 *re*lA1 and *E*. *coli strain* Top10 [F’[*lac*Iq Tn10(Tet^r^)] Δ *mcr*A Δ (*mrr hsd*RMS *mcr*BC) φ80*lac*Z ΔM15 Δ*lac*X74 *nup*G *rec*A1 *ara*D139 Δ (*ara- leu* 7697 galE15 *gal*K16 *rps*L (Str^r^) *end*A1 λ−] were grown in Luria-Bertani broth (LB) at 37°C or 30°C. *Salmonella enterica* sv. Typhimurium χ3987 [29] obtained from Roy Curtiss III was grown in Luria-Bertani broth (LB) in the presence of diaminopimelic acid (DAP) (100 μg/ml final concentration). *N*. *gonorrhoeae* strains FA1090 (obtained from Dr. W. Shafer at Emory University, Atlanta, GA), MS11 (obtained from Dr. H. Schneider at WRAIR, Washington, DC), F62 (obtained from Dr. P. F. Sparling, University of North Carolina, Chapel Hill, NC),1291B and FA1090 Δ NgoΦ6, ΔNgoΦ7, ΔNgoΦ8, ΔNgoΦ9 (from Dr DC Stein at the University of Maryland at College Park) were for these studies. *Neisseria* strains were grown in phosphate-buffered gonococcal medium (Difco) supplemented with 20 mM glucose and growth supplements [30] either in broth with the addition of 0.042% NaHCO_3_ or on agar at 37°C in an incubator with 5% CO_2_. Phagemid pBS::Φ6 construction and properties were described previously [24]

### Enzymes and chemicals

DNA and protein size markers were purchased from Fisher Scientific (Vilno). All chemicals used were reagent grade or better and were obtained from Sigma-Aldrich (St. Louis, MO), unless otherwise noted.

### Phage and phagemid particle preparation

Phage isolation was performed as previously described [20, 23–24] with some modifications. Overnight cultures were diluted 50-fold into 1000 ml of an appropriate medium and grown overnight with shaking at 30°C or 37°C. Bacteria were collected by centrifugation (20 min at 7,000 rpm.) The supernatant was mixed with 1/5 volume of a solution containing 20% polyethylene glycol (PEG-8000) and 2.5 M NaCl and kept at 4°C overnight to precipitate the phage particles. The precipitate was collected by centrifugation, dissolved in 4 ml of phosphate-buffered saline (PBS) and centrifuged at 4,000 rpm for 10 min. The phage particles were then purified by sequential centrifugation of the PBS phage suspension at 4,000 rpm for 10 min and 19,000 rpm for 120 min at 4°C in an SS34 rotor. The precipitate was dissolved in 1 ml of 50 mM Tris-HCl buffer, pH 7.5, and phage particles were further purified on a QMA-Sephacell column (2 cm by 10 cm) [24].

### Electron microscopy

Electron microscopy was carried out as described previously [24]. Overnight culture of *N*. *gonorrhoeae* FA1090 was grown in GC medium, diluted 30 times into fresh media, and incubated with shaking for 16 h. Phage particles were precipitated by the addition of NaCl to a final concentration of 1 M, polyethylene glycol 8000 to 10%. After collection of the precipitate by centrifugation, the precipitate was dispersed in SM buffer. The solution was centrifuged for 30 min at 12,500 rpm in a Beckman JA20 rotor at 4°C, the pellet was resuspended in Tris-EDTA (TE) buffer, and the solution was applied to a QMA Sephacell column (2 by 15 cm) (25). An aliquot of the eluate was stained with uranyl acetate (2%) for 30 s prior to visualization on a Zeiss EM10CA microscope (80 kV).

### Production of polyclonal antisera

Samples of purified NgoΦ6 particles containing 200 μg were used in EUROGENETIC S.A. Liege Belgium for immunization of three rabbits according to their standard protocol (ref. no AS-PNOR-3MORAB). In this protocol, phage suspensions were introduced three times subcutaneously at days 0, 14 and 28 in each animal per immunization without any adjuvant. Sera was collected at day 0 (before immunization) and at days 14 and 28. To determine the activity, these sera were pooled. All animal work performed at the EUROGENETIC S.A. Liege Belgium was carried out in accordance with the 2010/63/EU directive on the protection of animals used for scientific purposes. The protocols were approved under reference CE/SANTE/E/001 by the CER ethical licensing committee.

### Determination of antibodies against phage particles by dot spot ELISA

Determination of antibodies against phage particles was carried out as described previously [20] with modifications. NgoΦ6fil phage suspension (3 μl) containing protein at a concentration of 1,000 μg/ml in carbonate buffer (50 mM sodium bicarbonate, 0.03 M sodium azide, pH 9.6) was spotted in triplicate onto a nitrocellulose membrane and dried at room temperature. After three washes with 20 ml of PBS buffer, the membranes were blocked with 1% alkaline casein (Sigma) in PBS at 25°C for 1 h. The membranes were then washed three times for 20 min with PBS. Following the washes, different dilutions of sera collected after 0, 14 and 28 days in PBS with 1% alkaline casein was incubated at 25°C overnight. The membranes were washed three times with PBS and incubated with secondary antibodies at room temperature for 1 h (alkaline phosphatase-conjugated goat anti-rabbit IgG (ref. no M1421, Sigma-Aldrich, USA) diluted in PBS at 1:2,000). The secondary antibody was removed, and the membranes were washed four times for 15 min each with PBS + 0.1% Tween-20 and once with PBS. Membrane were soaked in 20 ml of detection buffer (AP, 0,1 M Tris-HCl pH 9,5; 0,1 M NaCl; 5 mM MgCl_2_, pH 9,5) containing 20 μl of NBT BCIP (Sigma, USA) for 30 min at room temperature in darkness. The reaction was stopped by intensive washing with distilled water and left to dry. The amount of protein contained in each spot visualized on the membrane and quantified using GeneTools GBox (Syngen) program. The intensity of each spot was expressed as the increase of the intensity compared to the negative control, where spotting of phage was omitted.

### Determination of the level of antibodies against *N*. *gonorrhoeae* cells by dot blot ELISA

Determination of the level of anti-*N*. *gonorrhoeae* specific IgG and IgA antibodies in rabbit sera was based on the method by Afonina *et al*. [31] and Cole and Jerse [32] and carried out as described previously in detail [20].

### Flow cytometric analysis

Flow cytometry was performed according to Price *et al*. [33] and described previously in detail in Piekarowicz *et al*. [20].

### Serum bacterial assay

The serum bacterial assay was essentially carried out according to Lin *et al*. [34] and described in detail previously by Piekarowicz *et al*. [20]. Gonococcal strains FA1090, F62 MS11 and 1291B were resuscitated on GC chocolate agar directly from a freezer stock. The plates were incubated at 37°C and 5% CO2 for 16 to 18 h, and the bacteria were resuspended in prewarmed GCK medium (37°C). The cell suspension was adjusted to approximately 3 × 10^4^ CFU/ml. Rabbit sera were pooled (n  =  3) and heat inactivated at 56°C for 30 min. A total of 50 μl of serially diluted serum samples in PBS and 40 μl of bacterial suspension were mixed and incubated at 37°C and 5% CO_2_ for 15 min. Undiluted normal baby rabbit sera (Sigma) (6 μl) was added to the mixtures to supply a complement source, and the incubation was continued for an additional 45 min. Samples were plated onto three plates of GCK agar and incubated for approximately 24 h, and colonies were enumerated. Titers were calculated as the reciprocal of the dilution that resulted in >50% killing compared to CFU detected in the presence of antibody but in the absence of baby rabbit sera. Assays were performed at least in triplicate. In all experiments, killing of bacteria by complement alone was less than 4%.

### Adherence assay

The adherence assay was carried out as described by Cole and Jerse [32]. In brief, ME 180 cervical epithelial cells were grown to near confluency in 24-well tissue plates in McCoy’s 5A medium ME180 cervical epithelial cells (ATCC, Manassas, VA) supplemented with 10% heat-inactivated fetal bovine serum (FBS) (Quality Biological Inc., Gaithersburg, MD) and 2.2 g/L sodium bicarbonate. *N*. *gonorrhoeae* bacteria were subcultured from the freezer and passed to GC agar before being suspended in McCoy’s 5A medium supplemented with 2.2 g/L sodium bicarbonate and 5 mg/L Kellogg’s solution containing 0.05% saponin to an OD_600_ of 1.0. The bacterial suspensions were then diluted to 10^6^ cells/ml and pre-incubated for 30 min with rabbit sera obtained after immunization with NgoΦfil phages. A total of 140 μl of bacterial suspension was applied to cells (multiplicity of infection, 10:1) in triplicate wells. After 3.5 h at 37°C in 5% CO_2_ monolayers, the cells were washed four times with PBS to remove no adherent bacteria. Cells were lysed with 0.5% saponin (Sigma), and the number of cell-associated bacteria was determined by plating the saponin-treated suspensions. The results are expressed as the number of cell-associated bacteria divided by the number of bacteria in the inoculum (% cell associated). The average percentage of cell-associated bacteria recovered from the test and control wells was calculated from three independent experiments that were each performed in triplicate. Standard error bars are shown.

### Statistical analysis

All statistical analysis was carried) using Stuout using Social Science Statistic (http://www.socstatistics.comdent’s *t* test. One-tailed *P* values of <0.05 –< 0.1 were considered statistically significant (see S6–S14 Fig).

## Supporting information

S1 Fig Original gel stained with Coomasie Brilliant Blue used for creation of Fig 1. Lines used in formation of Fig 1 are marked with asterisk.
(TIF)

S2 Fig The ORF4 and ORF5 phage proteins are the main structural proteins of phage NgoΦ6 and its phagemid derivatives. To construct the pBSNgoΦ6fm::ORF5::FLAG plasmid, pBSΦ6fm DNA was amplified using Orf5::FlagCFor 5’ GAC GAT GAC GAC AAG TGA TGG ATT TTT ATT TC 3 and Orf5::FlagCRev 5’ TTTGTA GTC TTT CAA AAC CTT TTT CAG CAG GG 3’ primers, and the resulting linear amplicon was cleaved with DpnI for 4 h at 37°C. After purification, the amplicon was treated with kinase, purified and ligated. The reaction products were purified and used for transformation of *E*. *coli* strain Top10. Recombinant phagemid particles pBS::NgoΦ6fm::ORF5::FLAG were obtained from one of the transformants. Phagemid particles were separated on SDS-PAGE gels and subjected to Western blot analysis with monoclonal anti-FLAG antibodies (DYKDDDDK Tag Monoclonal Antibody (FG4R) MA1-91878 Thermo Fisher Scientific, dilution 1:5,00 to 1:1,000), (lane 1). The antibodies show reactivity with two proteins derived from this phagemid particle. A protein with a molecular size of 12.5 kDa (lane b) corresponds to ORF5, and the 25 kDa protein (lane a) is formed by a fusion of the ORF4 and ORF5 proteins.
(TIF)

S3 Fig Original western used for creation of S2 Fig. Lines used in formation of S2 Fig. are marked with asterisk.
(TIF)

S4 Fig Original western used for creation of Fig 4. Lines used in formation of Fig 4 are marked with asterisk.
(TIF)

S5 Fig Original western used for creation of Fig 4. Lines used in formation of Fig 4 are marked with asterisk.
(TIF)

S6 Fig Determination of *P* value of antibodies present in GP sera binding *N*. g*onorrhoeae* cells. (DOCX)

S7 Fig Determination of *P* value of antibodies present in SAB sera binding *N. gonorrhoeae* cells. (DOCX)

S8 Fig Determination of *P* value of antibodies present in GP sera binding phage NgoΦ6 particles. (DOCX)

S9 Fig Determination of *P* value of *N. gonorrhoeae* F62 killing effect by SAB sera. (DOCX)

S10 Fig Determination of *P* value of *N. gonorrhoeae* 1291B killing effect by SAB sera. (DOCX)

S11 Fig Determination of *P* value of *N. gonorrhoeae* FA1090 killing effect by SAB sera. (DOCX)

S12 Fig Determination of *P* value for blocking of adherence of *N. gonorrhoeae* FA1090 to eukaryotic cells by antibodies present in SAB sera. (DOCX)

S13 Fig Determination of *P* value for blocking of adherence of *N. gonorrhoeae* MS11 to eukaryotic cells by antibodies present in SAB sera. (DOCX)

S14 Fig Determination of *P* value for blocking of adherence of *N. gonorrhoeae* F62 to eukaryotic cells by antibodies present in SAB sera. (DOCX)

## References

[1] Curtiss R III. Bacterial infectious diseases control by vaccine development. The Journal of Clinical Investigation. 2002;110: 1061–1066 10.1172/JCI16941 12393839 PMC150804

[2] Barney S, Graham CS. Advances in Antiviral vaccine development Immunol Rev. 2013; 255(1): 10.1111/imr.12098 23947359 PMC3821995

[3] Ferraro B, Morrow MP, Hutnick NA, Shin TS, Lucke C.E., David B, et al, Clinical Applications of DNA Vaccines: Current Progress. Clin Infect Dis.2011; 53(3): 296–302. 10.1093/cid/cir334 21765081 PMC3202319

[4] Ingolotti M, Kawalekar O, Shedlock D. Muthumari K., Werner D. Expert on Vaccines 9,747–763 (2010) 10.1586/en10.57 PMC2962930 20624048

[5] Lindquist B. H. Phage Display In Calender R (ed.), Bacteriophages. (Oxford University Press, 2006).

[6] Gaubin M. et al Processing of filamentous bacteriophage virions in antigen- presenting cells targets both HLA class I and class II peptide loading compartments. DNA Cell Biol. 2003 22, 11–18 (2003). 10.1089/104454903321112451 12590733

[7] Hashemi H., Bamdad T., Jamali A., Pouyanfard S. Mohammadi M. G. Evaluation of humoral and cellular immune responses against HSV-1 using genetic immunization by filamentous phage particles: A comparative approach to conventional DNA vaccine. J. Virol. Meth. 163, 440–444 (2010). 10.1016/j.jviromet.2009.11.008 19903497

[8] Ulivieri C., Citro A, Ivaldi F, Mascolo D, Ghittoni R, Fanigliulo D et al Antigenic properties of HCMV peptides displayed by filamentous bacteriophages vs. synthetic peptides. Immunol. Lett. 119, 62–70 (2008). 10.1016/j.imlet.2008.04.004 18538862

[9] Wan Y. Wu Y, Zhou J, Zou L, Liang Y, Zhao J, Cross-presentation of phage particle antigen in MHC class II and endoplasmic reticulum marker-positive compartments. Eur. J. Immunol. 35, 2041–2050 (2005). 10.1002/eji.200425322 15940671

[10] De Berardinis P, Haigwood NL. New recombinant vaccines based on the use of prokaryotic antigen-display systems. Exp. Rev. Vac. 3, 673–679 (2004). 10.1586/14760584.3.6.673 15606352

[11] Manoutcharian K., Gevorkian G., Cano A. & Almagro J. C. Phage displayed biomolecules as preventive and therapeutic agents. Curr. Pharm. Biotech. 2, 217–223 (2001). 10.2174/1389201013378671 11530876

[12] Samoylova T. I. et al Infective and inactivated filamentous phage as carriers for immunogenic peptides. J. Virol. Meth. 183, 63–68 (2012). 10.1016/j.jviromet.2012.03.032 22575687

[13] Grabowska A. M. et al Immunisation with phage displaying peptides representing single epitopes of the glycoprotein G can give rise to partial protective immunity to HSV-2. Virol. 269, 47–53 (2000). 10.1006/viro.2000.0185 10725197

[14] Parren P. W. & Burton D. R. Antibodies against HIV-1 from phage display libraries: mapping of an immune response and progress towards antiviral immunotherapy. Chem. Immunol. 65, 18–56 (1997). 9018871 10.1159/000319346

[15] Frenkel D., Dewachter I., Van Leuven F. & Solomon B. Reduction of beta- amyloid plaques in brain of transgenic mouse model of Alzheimer’s disease by EFRH-phage immunization. Vaccine 21, 1060–1065 (2003). 12559780 10.1016/s0264-410x(02)00609-6

[16] Eriksson F. et al Tumor specific phage particles promote tumor regression in a mouse melanoma model. Cancer Immunol. Immunother. 56, 677–687 (2007). 10.1007/s00262-006-0227-6 16967280 PMC11031031

[17] Balcioglu B. K., Ozdemir-Bahadir A., Hinc D., Tamerler C. & Erdag B. Cost effective filamentous phage based immunization nanoparticles displaying a full-length hepatitis B virus surface antigen. Adv. Biosci. Biotech. 5, 46–53 (2014).

[18] Sartorius R. et al Vaccination with filamentous bacteriophages targeting DEC- induces DC maturation and potent anti-tumor T-cell responses in the absence of adjuvants. Eur. J. Immunol. 41,2573–2584 (2011). 10.1002/eji.201141526 21688262

[19] Krag DN, Shukla GS, Shen GP, Pero S, Ashikaga T, Fuller S, et al Selection of tumor-binding ligands in cancer patients with phage display libraries. Cancer Res. 66, 7724–7733 (2006). 10.1158/0008-5472.CAN-05-4441 16885375

[20] Piekarowicz A, Kłyż A, Majchrzak M, Stein DC. Immunization of Rabbits with S. enterica Typhimurium Expressing Neisseria gonorrhoeae Filamentous Phage Φ6 Induces Bactericidal Antibodies Against N. gonorrhoeae. Sci Rep. 2016 3 4;6:22549 10.1038/srep22549 Free PMC Article 26939573 PMC4778046

[21] Jerse AE, Bash MC, Russell MW. Vaccines against gonorrhea: current status and future challenges. Vaccine 32, 1579–1587 (2014). [PMC free article] 10.1016/j.vaccine.2013.08.067 24016806 PMC4682887

[22] Kawai M, Uchiyama I, Kobayashi J. Genome comparison in silico in *Neisseria* suggests integration of filamentous bacteriophages by their own transposase. DNA Res. 12, 389–401 (2005). 10.1093/dnares/dsi021 16769696

[23] Piekarowicz A, Majchrzak M, Kłyż A, Adamczyk-Poplawska M. Analysis of the filamentous bacteriophage genomes integrated into *Neisseria gonorrhoeae* FA1090 chromosome. Pol. J. Microbiol. 2006;55: 251–260. 17416061

[24] Piekarowicz A, Kłyż A, Majchrzak M, Szczêsna E, Piechucki M, Kwiatek A, Maugel TK, et al Neisseria gonorrhoeae filamentous phage NgoPhi6 is capable of infecting a variety of Gram-negative bacteria. J. Virol. 2014; 88: 1002–1010. [PMC free article] 10.1128/JVI.02707-13 24198404 PMC3911633

[25] Meyer J, Brissac T, Frapy E, Omer H, Euphrasie D, Bonavita AE, et al Characterization of MDAΦ, a temperate filamentous bacteriophage of Neisseria meningitidis. Microbiology. 2016 162(2):268–82. 10.1099/mic.0.000215 26602366

[26] Kevin AH, Arbabi-Ghahroudi M, Scott JK. Beyond phage display: non- traditional applications of the filamentous bacteriophage as a vaccine carrier, therapeutic biologic, and bioconjugation scaffold. Front. Microbiol. 2015; 10.3389/fmicb.00755 PMC4523942 26300850

[27] Russel M, Model P. Filamentous phages(ed.) Oxford University Press, (2006)

[28] Cain JA, Solis N, Cardwell SJ. Beyond gene expression: The impact of protein post-translational modifications in bacteria. J. Proteomics 2014; 97: 265–286. 10.1016/j.jprot.2013.08.012 23994099

[29] Galan JE, Nakayama K, Curtiss R. 3rd. Cloning and characterization of the asd gene of *Salmonella typhimurium*: use in stable maintenance of recombinant plasmids in *Salmonella* vaccine strains. 1990; Gene 94; 29– 35. 2227450 10.1016/0378-1119(90)90464-3

[30] White LA, Kellogg DS Jr. *Neisseria gonorrhoeae* identification in directsmears by a fluorescent antibody counterstain method. Appl. Microbiol.1965;13: 171–174. 14325874 10.1128/am.13.2.171-174.1965 PMC1058216

[31] Afonina G, Leduc I, Nepluev I, Jeter C, Routh P, Almond G, Orndorff PE, et al Immunization with the *Haemophilus ducreyi* hemoglobin receptor HgbA protects against infection in the swine model of chancroid. Infect. Immun.2006; 74; 2224–2232. 10.1128/IAI.74.4.2224-2232.2006 16552053 PMC1418891

[32] Cole JG, Jerse AE. Functional characterization of antibodies against *Neisseria gonorrhoeae* opacity protein loops. 2009 PLoS One 4, e8108 10.1371/journal.pone.0008108 19956622 PMC2779592

[33] Price G A, Masri H P, Hollander AM, Russell MW, Cornelissen CN. Gonococcal transferrin binding protein chimeras induce bactericidal and growth inhibitory antibodies in mice. Vaccine 25, 7247–7260 (2007) 10.1016/j.vaccine.2007.07.038 17720283 PMC2225598

[34] Lin M, Todoric D, Mallory M, Luo BS, Trottier E, Dan H et al Monoclonal antibodies binding to the cell surface of Listeria monocytogenes serotype 4b. J. Med. Microbiol.55, 291–299 (2006). 10.1099/jmm.0.46305-0 16476793

