# Supplementary figures and images for: Phage proteins are expressed on the surface of Neisseria gonorrhoeae and are potential vaccine candidates
Source: PLoS One. 2018 Aug 23;13(8):e0202437. doi: 10.1371/journal.pone.0202437 (PMC6107182; doi:10.1371/journal.pone.0202437)

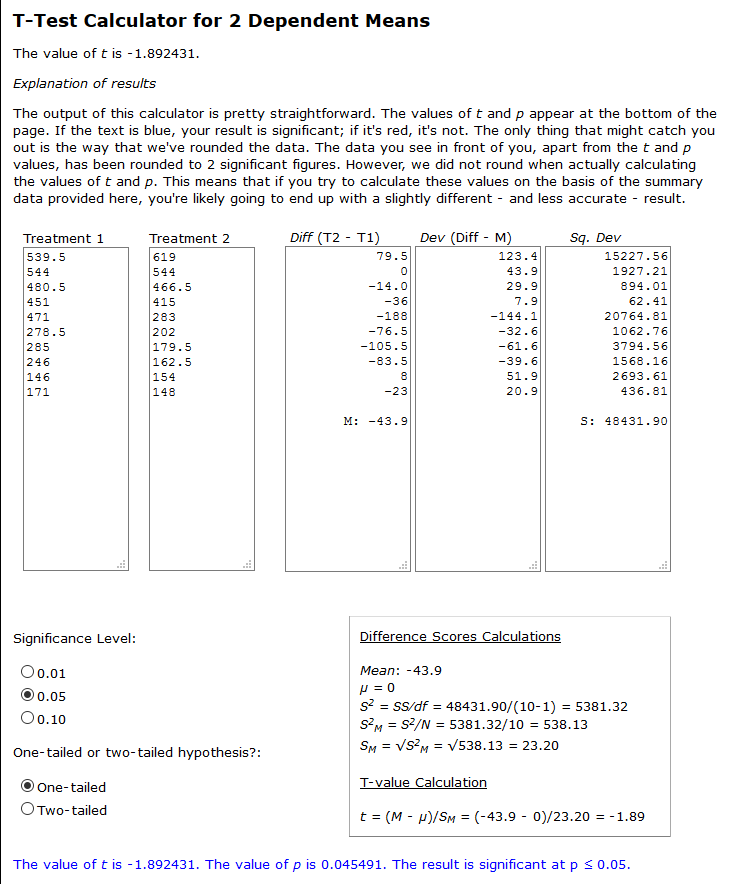

Supplement: S6 Fig — (DOCX) [file pone.0202437.s006.docx]

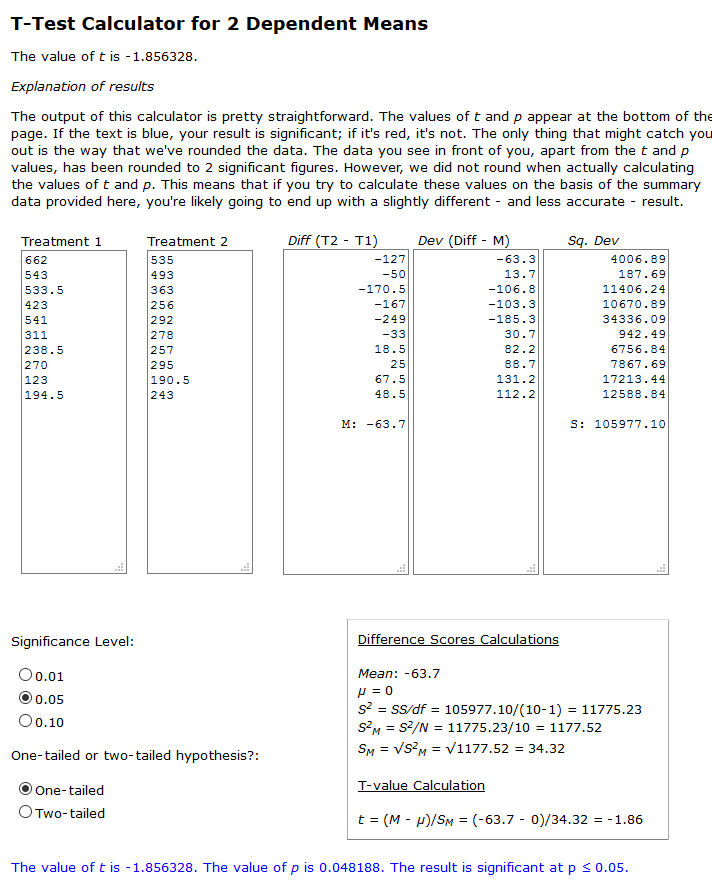

Supplement: S7 Fig — (DOCX) [file pone.0202437.s007.docx]

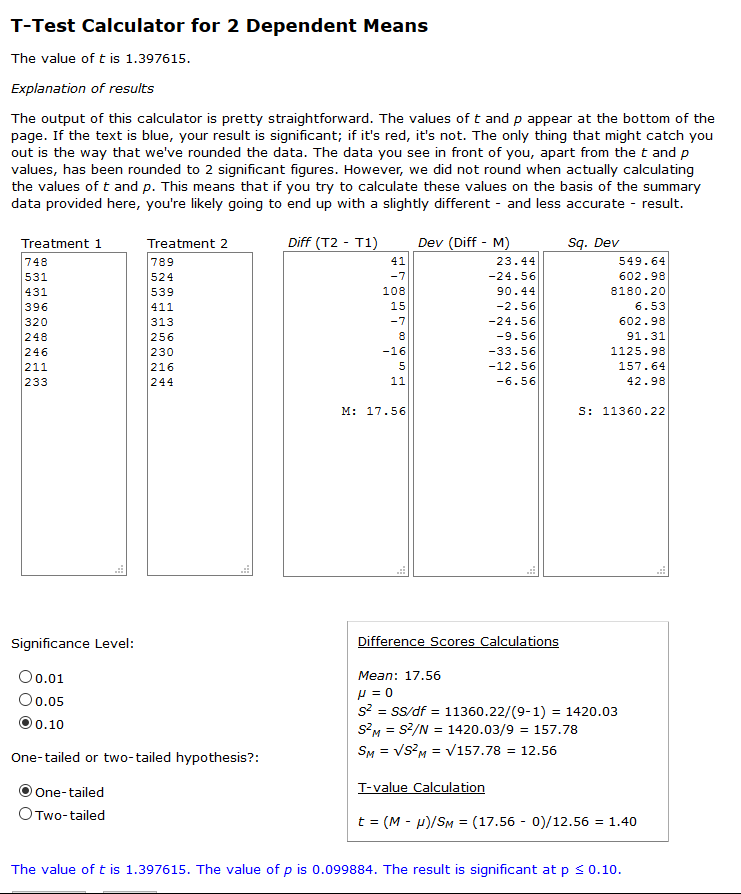

Supplement: S8 Fig — (DOCX) [file pone.0202437.s008.docx]

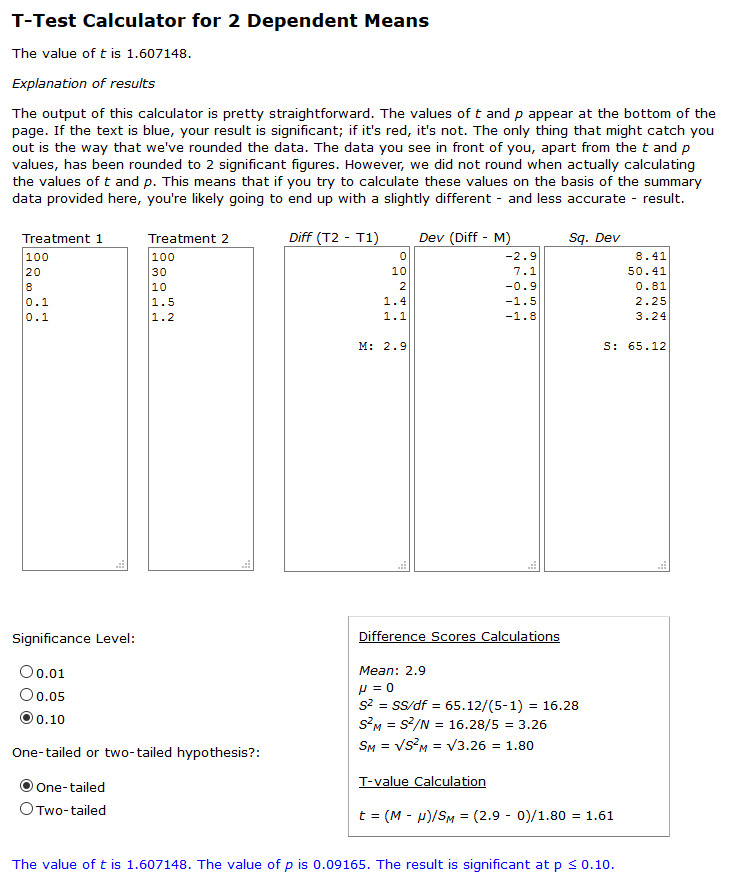

Supplement: S9 Fig — (DOCX) [file pone.0202437.s009.docx]

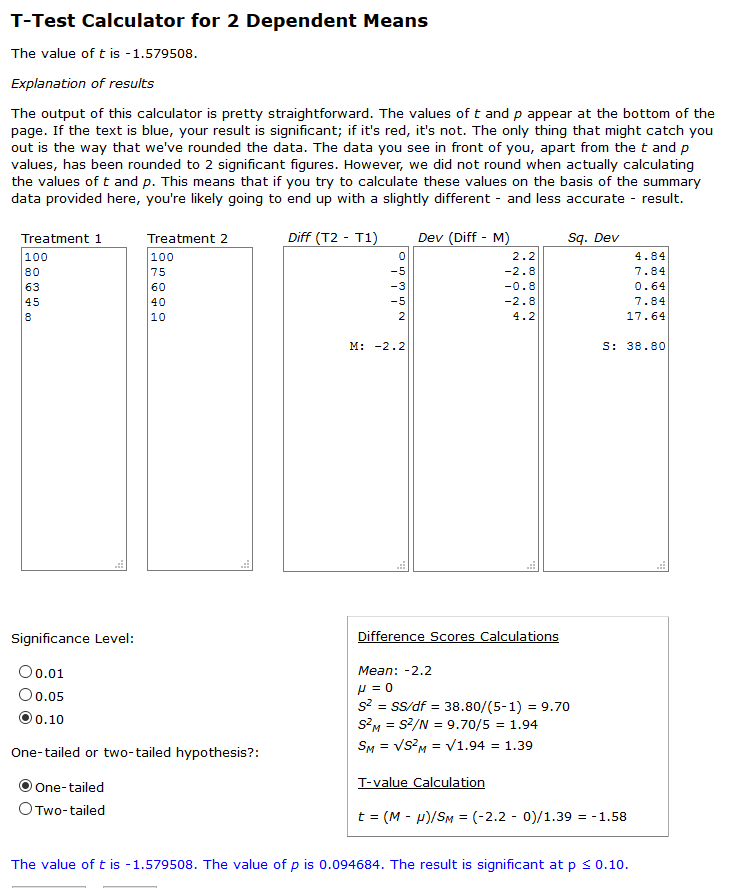

Supplement: S10 Fig — (DOCX) [file pone.0202437.s010.docx]

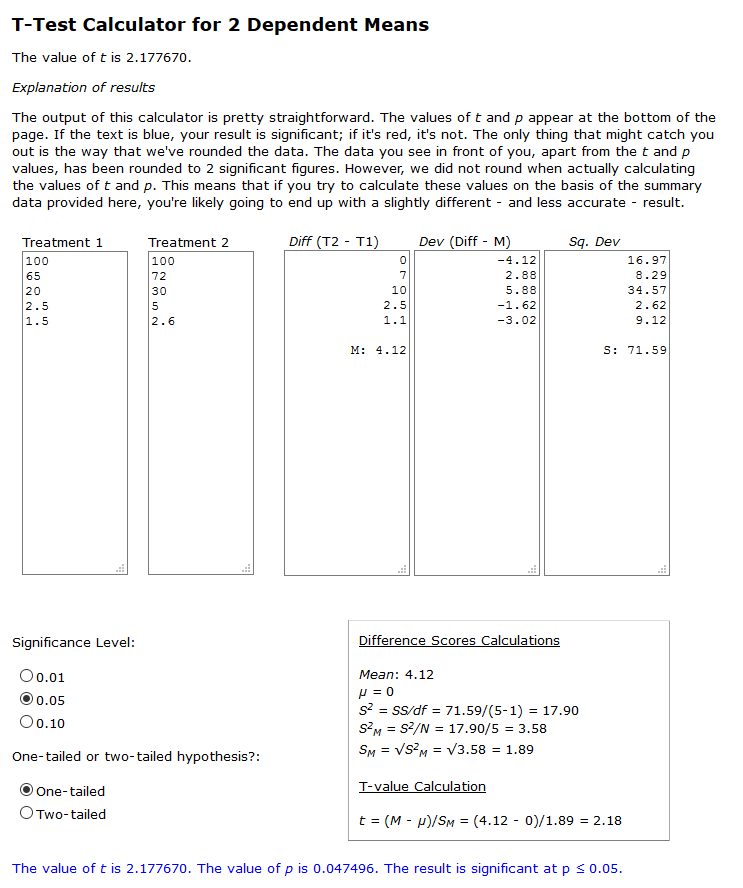

Supplement: S11 Fig — (DOCX) [file pone.0202437.s011.docx]

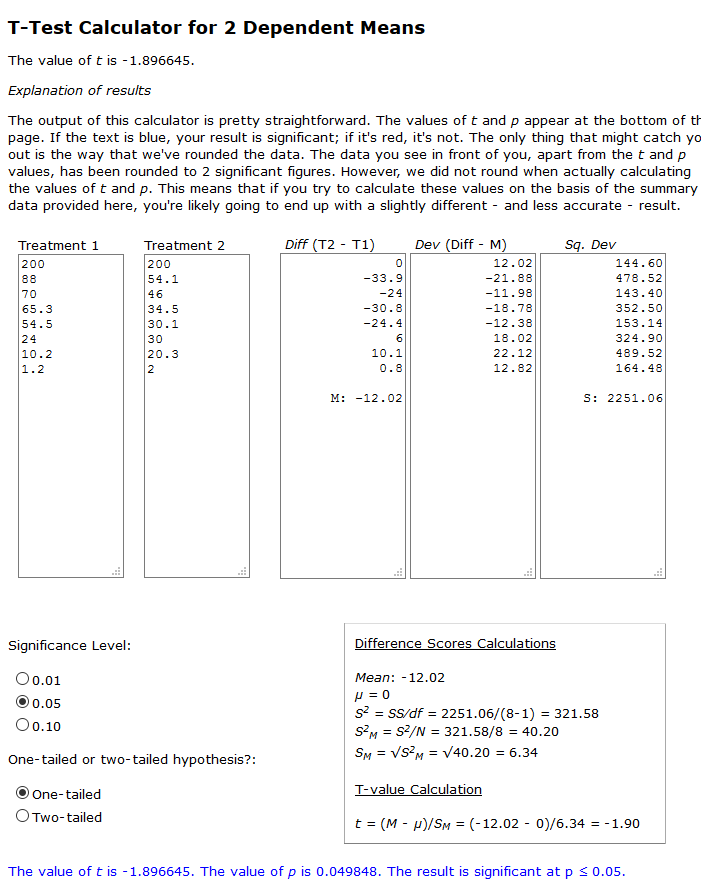

Supplement: S12 Fig — (DOCX) [file pone.0202437.s012.docx]

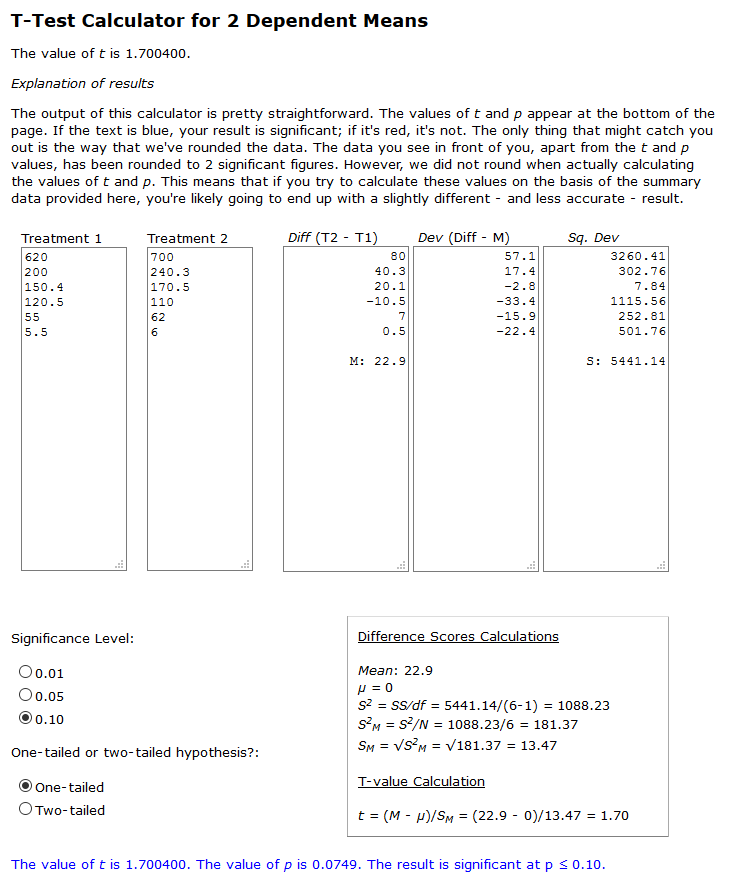

Supplement: S13 Fig — (DOCX) [file pone.0202437.s013.docx]

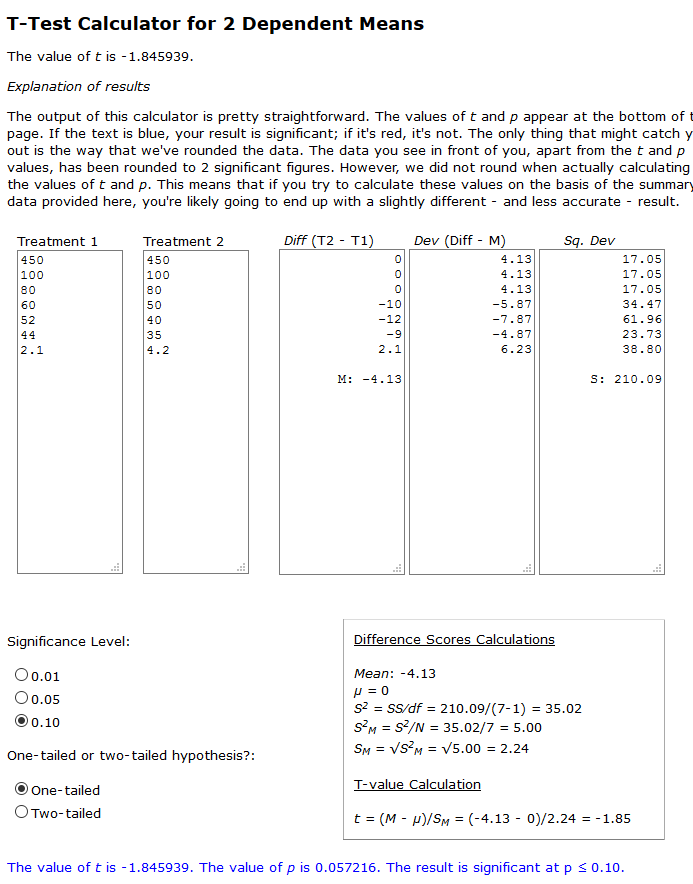

Supplement: S14 Fig — (DOCX) [file pone.0202437.s014.docx]
